# Intradermal Immunization of EBOV VLPs in Guinea Pigs Induces Broader Antibody Responses Against GP Than Intramuscular Injection

**DOI:** 10.3389/fmicb.2020.00304

**Published:** 2020-02-27

**Authors:** Ying Liu, Zhiyuan Wen, Ricardo Carrion, Jerritt Nunneley, Hilary Staples, Anysha Ticer, Jean L. Patterson, Richard W. Compans, Ling Ye, Chinglai Yang

**Affiliations:** ^1^State Key Laboratory of Food Nutrition and Safety, Institute of Health Biotechnology, College of Biotechnology, Tianjin University of Science and Technology, Tianjin, China; ^2^Department of Microbiology and Immunology and Emory Vaccine Center, School of Medicine, Emory University, Atlanta, GA, United States; ^3^Harbin Veterinary Research Institute, Harbin, China; ^4^Texas Biomedical Research Institute, San Antonio, TX, United States

**Keywords:** ebola, vaccine, intradermal immunization, antibody response, VLP

## Abstract

Ebolavirus (EBOV) infection in humans causes severe hemorrhagic fevers with high mortality rates that range from 30 to 80% as shown in different outbreaks. Thus the development of safe and efficacious EBOV vaccines remains an important goal for biomedical research. We have shown in early studies that immunization with insect cell-produced EBOV virus-like particles (VLPs) is able to induce protect vaccinated mice against lethal EBOV challenge. In the present study, we investigated immune responses induced by Ebola VLPs via two different routes, intramuscular and intradermal immunizations, in guinea pigs. Analyses of antibody responses revealed that similar levels of total IgG antibodies against the EBOV glycoprotein (GP) were induced by the two different immunization methods. However, further characterization showed that the EBOV GP-specific antibodies induced by intramuscular immunization were mainly of the IgG2 subtype whereas both IgG1 and IgG2 antibodies against EBOV GP were induced by intradermal immunization. In contrast, antibody responses against the EBOV matrix protein VP40 induced by intramuscular or intradermal immunizations exhibited similar IgG1 and IgG2 profiles. More interestingly, we found that the sites that the IgG1 antibodies induced by intradermal immunizations bind to in GP are different from those that bind to the IgG2 antibodies induced by intramuscular immunization. Further analyses revealed that sera from all vaccinated guinea pigs exhibited neutralizing activity against Ebola GP-mediated HIV pseudovirion infection at high levels. Moreover, all EBOV VLP-vaccinated guinea pigs survived the challenge by a high dose (1000 pfu) of guinea pig-adapted EBOV, while all control guinea pigs immunized with irrelevant VLPs succumbed to the challenge. The induction of both IgG1 and IgG2 antibody responses that recognized broader sites in GP by intradermal immunization of EBOV VLPs indicates that this approach may represent a more advantageous route of vaccination against virus infection.

## Introduction

Ebolavirus is a member of the filoviridae family, and infection by ebolavirus in humans and non-human primates (NHPs) results in onset of severe hemorrhagic fevers with high mortality rates (Feldmann and Geisbert, 2011; Li H. et al., 2015; Ye and Yang, 2015). Since their first identification in 1976, five different ebolavirus species have been isolated from outbreaks in humans or NHPs including Ebola virus (EBOV), Sudan virus (SUDV), Bundibugyo virus (BDBV), Tai Forest virus (TAFV), and Reston virus (RESTV), and these viruses differ significantly in their amino acid sequences by as much as 40% (Towner et al., 2008). Notably, the RESTV has been found only in non-human primates from the Philippines, whereas the other four filovirus species are only detected in tropical areas of the equator Africa, and studies in recent years have shown that the African green fruit bats may serve as the natural reservoir of these ebolaviruses (Leroy et al., 2005; Groseth et al., 2007). It is noted that in recent years ebolavirus infection of humans has become more frequent (Chowell and Nishiura, 2014). Of particular concern, the 2013–2016 EBOV outbreak that infected over 28000 human infections and resulted in over 11000 deaths. Moreover, the current ongoing 2018 Kivu outbreak has a mortality rate around 67% and has thus far resulted in over 2000 individuals killed. These large-scale outbreaks demonstrates that the serious threat of EOBV infection to public health is urgent and real. Furthermore, evidence also suggests that EBOV may also infect dogs during outbreaks in addition to infect humans and NHPs (Allela et al., 2005). On the other hand, RESTV has also been indicated to infect domestic pigs in Asia based on serological analyses (Barrette et al., 2009). Further, EBOV has been demonstrated to infect pigs in experimental settings with causing pathogenesis, and virus from infected pigs were shown to transmit to NHPs with no direct contact, demonstrating that this highly lethal virus may be capable of aerosol transmission from a infected host to another susceptible host (Reed et al., 2011; Weingartl et al., 2012). The potential of EBOV to cause non-pathogenic infection in domestic pigs poses a grave danger for these viruses to become endemic and infect humans through zoonotic transmission.

The high mortality rate of EBOV infection underscores the urgent need for an effective EBOV vaccine. A number of EBOV vaccine approaches have been explored in past studies, many of these vaccine strategies have been shown to be able to protect vaccinated animals against lethal EBOV challenge in small laboratory animal models with various efficacies (Yang et al., 2008; Marzi and Feldmann, 2014). Moreover, promising results have also been obtained with viral vector-based vaccine strategies in the NHP model for protection against lethal EBOV challenge, which include recombinant adenovirus replicons (Sullivan et al., 2006), VSV (Jones et al., 2005), parainfluenza virus (Bukreyev et al., 2007), Rabies virus (Blaney et al., 2013), VRP (Herbert et al., 2013), as well as a replication defective EBOV (Marzi et al., 2015) vaccines. In addition to viral vector-based vaccines, EBOV virus-like particles (VLPs) and other protein-based subunit vaccines have been shown to induce protective immune responses against lethal EBOV infection of NHPs (Warfield et al., 2007; Swenson et al., 2008). Of these, recombinant adenovirus replicons and recombinant VSV based vaccines have been evaluated in a number of Phase I clinical trials and shown to be safe and immunogenic (Ewer et al., 2016; Regules et al., 2017). More recently, a Phase III clinical trial was conducted in Guinea with a the recombinant VSV-based vaccine that expresses EBOV GP, and this vaccine was found to be highly effective in preventing EBOV infection of people with high risks to EBOV infections (Henao-Restrepo et al., 2015). The success of the recombinant VSV/EBOV vaccine in the Phase III trial showed that an effective vaccine can control the EBOV epidemics. Nonetheless, the efficacy of viral vector-based EBOV vaccines may be hampered by the pre-existing immunity in the affected area to these viral vectors. Further, immunization by such vaccines may also induce strong immune responses against the vector and therefore dampen their ability to induce immune responses in boosting immunizations for achieving durable protection or in subsequent vaccination of high risk personnel against another filovirus in future outbreaks. Thus, development of vaccine strategies to overcome the limitations of viral vector-based vaccines is still in need.

Among the different EBOV vaccine strategies, VLPs are a subunit vaccine platform that has been shown to protect EBOV infection in NHPs. In early studies, both EBOV and Marburg virus VLPs have been evaluated in different animal models and shown to be effective in eliciting protecting vaccinated animals against lethal challenge (Warfield and Aman, 2011), demonstrating that this vaccine strategy is able to control and prevent filovirus infection. We have shown in our previous studies that EBOV VLPs produced in insect cells by the recombinant baculovirus expression system are able to stimulate dendritic cells to secret various cytokines, and neutralizing antibodies against infection mediated by the EBOV GP are induced in vaccinated animals immunized with these VLPs (Ye et al., 2006). We further demonstrated that immunization with the EBOV VLP vaccines produced from insect cells completely protected mice against lethal challenge by a high dose of mouse-adapted EBOV (Sun et al., 2009). In this study, we investigated immune responses elicited by EBOV VLPs in guinea pigs via different routes, through intramuscular (IM) and intradermal (ID) injection respectively, and showed that immunization by EBOV VLPs produced in insect cells effectively induced neutralizing antibodies against EBOV GP and conferred complete protection against lethal challenge by guinea pig-adapted EBOV. Further, we found that by IM immunization, the EBOV VLPs induced both IgG1 and IgG2 antibodies against EBOV GP in guinea pigs, as compared to the dominant IgG2 antibody responses induced by IM immunizations. More interestingly, we show that the IgG1 antibodies induced by ID immunizations against GP bind to different targets from those recognized by the IgG2 antibodies induced by IM immunizations, demonstrating the potential advantage of ID immunization in eliciting broader antibody responses against infection by EBOV.

## Materials and Methods

### Virus and Biosafety

The stock for guinea pig-adapted EBOV was produced and titered in Vero E6 cells. All experiments with live EBOV were conducted in the BSL-4 facility at the Texas Biomedical Research Institute (TxBiomed), San Antonio, TX, United States.

### Cells and Antibodies

*Spodoptera frugiperda* Sf9 cells were maintained in SF-900 II serum-free medium with penicillin/streptomycin. The polyclonal rabbit serum against EBOV was a gift from Dr. P. Rollin (CDC). JC53BL cells (Wei et al., 2003), which express β-galactosidase and luciferase under a *tat-*activated promoter, was obtained from NIH AID Reagents and Reference Program, and maintained in DMEM plus 10% fetal calf serum (FCS). HeLa cells and 293T cells were obtained from ATCC and maintained in DMEM plus 10% FCS.

### Production and Characterization of Ebola VLPs Produced in Insect Cells

Construction and production of recombinant baculoviruses (rBVs) that express EBOV GP and VP40 (designated as rBV-GP and rBV-VP40 respectively) has been described previously (Ye et al., 2006; Sun et al., 2009). For VLP production, Sf9 cells were grown in suspension and then infected with by rBV-GP and rBV-VP40 at the MOIs (multiplicity of infection) of 5 and 2 per cell for each virus. Cell culture medium was harvested at 48 h post-infection and clarified by centrifugating at 1500 RPM for 10 min in a 50 ml conical tube, and the supernatant was then concentrated through Quickstand filtration system (GE). Subsequently, VLPs were further purified through a discontinuous sucrose gradient (10–50%), concentrated again by ultra-centrifugation, and then resuspended in PBS with a concentration of 1 ug/ul (protein/volume) (Ye et al., 2006). Purified EBOV VLPs were analyzed by Western blot using rabbit serum against EBOV to detect viral VP40 and GP proteins, analyzed by electron microscopy to examine the morphology and integrity of VLPs, and analyzed by quantitative enzyme-linked immunosorbent assay (ELISA) to quantify the levels of GP proteins in VLP preparations (Sun et al., 2009). As a control antigen for vaccination, SIVgag VLPs were produced by expressing the SIV Gag protein in Sf9 cells using the recombinant baculovirus expression system and SIVgag VLPs were purified through a sucrose gradient similarly as described above and characterized as shown in previous studies (Ye et al., 2006).

### Immunization of Guinea Pigs and Sample Collection

Female Hartley guinea pigs (∼250 g in body weight) were obtained from the Charles River Laboratory and housed at the Division of Animal Research of Emory University. Twelve guinea pigs were divided into three groups with four animals per group. Two groups of guinea pigs were vaccinated by IM or ID injection first with 50 ug of EBOV VLPs two times and then boosted again with 200 ug EBOV VLPs at 4-week intervals. The control group guinea pigs were vaccinated with SIVgag VLPs (*n* = 4) of the same dose as an irrelevant VLP control. For ID injection, VLPs were delivered by using the Mantoux method to four sites (25 to 50 ul per site based on vaccine dose) of the shaved posterior-abdomen skin of guinea pigs under kentamine/xylazine anaesthetization. Blood samples were collected from cranial vena cava under anesthesia at 1 week prior to the first immunization and 2 weeks after the second and third immunizations, and stored at −80°C until being used in analyses.

### Detection of GP and VP40 Antigen-Specific Antibodies

Antibodies against EBOV GP and VP40 antigens were detected in serum samples from each guinea pig by ELISA. The His-tagged EBOV GP was produced by infecting HeLa cells with a recombinant vaccinia virus that expresses the EBOV GP-histag protein, and the GP-histag protein secreted into the medium of infected HeLa cells was purified using a Ni-NTA agarose bead-based higtag protein purification kit following established protocols (Sun et al., 2009). The His-tagged EBOV VP40 (VP40-histag) was expressed in bacteria DH5a using plasmid DNA vector pBlusscript IIKS under the T7 promoter, and purified using Ni-NTA agarose bead-based histag protein purification kit following manufacturer’s protocols. For ELISA analysis of serum samples, a microtiter plate (Maxisorb, Nunc) was coated overnight at 4°C with purified His-tagged EBOV GP or VP40 at a concentration of 2 ug/ml. Plates were then washed with PBST prior to blocking with 200 ul per well 2% BSA/PBST for 1 h at 37°C. Serum samples from vaccinated guinea pigs were serially diluted and then added to the wells of the microtiter plates that were coated and blocked and then incubated at room temperature for 2 h, and the bound antibodies specific for EBOV proteins were detected with horseradish peroxidase-labeled goat against guinea pig IgG, IgG1, or IgG2 antibodies (Bethyl Lab, Inc.). The wells were then washed and added with TMB (Sigma) at 50 ul per well to develop color, and the enzymatic reaction was stopped with addition of 50 ul per well of hydrochloric acid (0.2N), and afterward the absorbance value at 450 nm of each well was read in an ELISA reader (Bio-Tek Instruments, Inc., Winooski, VT, United States). Serial dilutions of purified guinea pig IgG (EQUITECH-BIO, Inc.) with known concentrations were used to generate a standard curve, which was used to calculate the concentrations of EBOV GP or VP40-specific antibodies in serum samples and the antibody concentrations of these antibodies were expressed as the amount of antibodies in 1 ml of serum (ng/ml).

### Blocking ELISA

Blocking ELISA was employed to investigate whether IgG2 antibodies induced by IM injection of EBOV VLPs will affect binding of IgG1 antibodies induced by ID injection of EBOV VLPs to GP. Briefly, microtiter plates were coated o/n at 4°C with purified His-tagged GP as coating antigens at a reduced concentration of 0.5 ug/ml. Plates were then washed with PBST prior to blocking with 200 ul per well 2% BSA/PBST for 1 h at 37°C, and then added with 1:400 dilution of sera from each individual guinea pig vaccinated by IM injection of EBOV VLPs, sera from naïve guinea pigs, or PBST only for 2 h at room temperature. This serum dilution (1:400) has been pre-determined to saturate binding of the amount of coated His-tagged GP antigens. The plates were then washed again and then incubated with sera from individual guinea pigs that had been vaccinated by ID injection of EBOV VLPs or SIVgag VLPs (Control). After incubation for 2 h at room temperature, the plates were washed and the bound IgG1 antibodies were detected with horseradish peroxidase-labeled goat against guinea pig IgG1 secondary antibodies (Bethyl Lab, Inc.). The wells were added with 50 ul per well of TMB (Sigma) to develop color and the color reaction was stopped with addition of 50 ul per well of hydrochloric acid (0.2N), and afterward the absorbance value at 450 nm of each well was read in an ELISA reader (Bio-Tek Instruments, Inc., Winooski, VT, United States).

### Analysis of Sera Neutralizing Activity With Pseudovirus

The levels of neutralizing antibodies against EBOV GP were detected by a single-round infectivity assay that we have developed in early studies (Mohan et al., 2012, 2015). Briefly, EBOV GP-HIV pseudovirions were produced by transfecting 293T-cells with and HIV backbone plasmid (Env-defective) and together with a DNA plasmid expressing EBOV GP in pCAGGS using the transfection reagent Fugene HD (Roche). At 48 h post-transfection, pseudovirions in the medium from transfected cells were harvested, clarified, and filtered using a 0.45 micron filter, and then titrated in JC53BL cells (Wei et al., 2003), which express β-galactosidase and luciferase under a *tat-*activated promoter. Neutralization assays were then carried out as described in our previous studies (Li W. et al., 2015). Briefly, pseudoviruses were fisrt incubated with serial dilutions of heat-inactivated serum samples for 1 h at 37°C, which were supplemented with heat-inactivated sera from naïve mouse (Innovative Research) to 5% of the total volume. The serum-pseudovirion mixtures were then added to JC53 cells that have been grown to 50% confluenvy in a 96-well plate and incubated at 37°C for 48 h. Afterward, the level of luciferase activity in each well were detected by a kit (Sigma), neutralization was calculated by measuring the percent decrease in luciferase activity in sample wells compared to virus-only control wells. The serum neutralizing activity is calculated with the formula: [(luciferase activity in control well – luciferase activity in sample well)/luciferase activity in control well × 100%], and is expressed as the percentage reduction of virus titers in sample wells compared to the titers in control wells. Statistical analysis of serum antibody responses between ID and IM vaccinated guinea pigs was performed by a Student’s *t*-test.

### Lethal Challenge by Guinea Pig-Adapted EBOV

EBOV challenge study was conducted in the ABSL-4 facility at Texas Biomedical Research Institute (San Antonio, TX, United States). Guinea pigs were challenged at 22 weeks after the third vaccination by intraperitoneal injection with 1000 plaque-forming units (pfu) of guinea pig-adapted EBOV. After challenge, guinea pigs were monitored at least twice daily for weight loss and disease symptoms, and animals that exhibited severe disease signs and lost significant levels of body weight (over 25%) were sacrificed in compliance with IACUC guidelines.

## Results

### Comparison of Antibody Responses Induced by EBOV VLPs Through Intradermal and Intramuscular Immunization Routes

**FIGURE 1:**
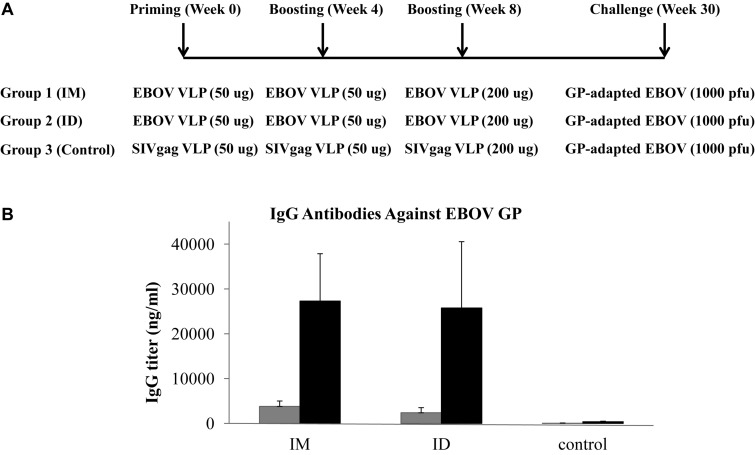
**(A)** Schematic diagram of immunization and challenge study design. Female Hartley guinea pigs (groups of 4) were vaccinated with EBOV VLPs or control SIVgag VLPs at 4-week intervals via different immunization routes as presented. Blood samples were collected at 2 weeks after the second and third immunizations and guinea pigs were challenged at 22 weeks after the final immunization by 1000 pfu of guinea pig-adapted EBOV. **(B)** Antibody responses against GP induced by IM or ID injection of EBOV VLPs. The levels of antibody response against EBOV GP induced by VLP vaccines in guinea pigs were measured by ELISA using purified His-tagged GP as coating antigen. Antibody concentration was determined from a standard curve and expressed as ng/ml of IgG against GP in serum samples collected after the second and third immunizations. Results reported are the means and standard deviations for samples from individual animals of each group. Gray columns, antibody levels in sera from vaccinated collected at 2 weeks after the second immunization; black columns, antibody levels in sera from vaccinated collected at 2 weeks after the third immunization.

In our previous studies, we showed that mice were effectively protected by two intramuscular immunizations with 50 ug EBOV VLPs produced in insect cells (Sun et al., 2009). To investigate the immunogenicity of insect cell-produced EBOV VLPs in guinea pigs via different immunization routes, we first vaccinated guinea pigs (groups of 4) with 50 ug EBOV VLPs by IM or ID injection as outlined in Figure 1A. The control group animals received immunization by 50 ug SIVgag VLPs that were similarly produced in insect cells by IM or ID injection (2 animals each route). As outlined in Figure 1A, blood samples were first collected at 2 weeks after the second immunization and then analyzed for antibodies against EBOV GP. As shown in Figure 1B (gray columns), antibodies against EBOV GP were readily detected in sera from guinea pigs after two immunizations with 50 ug EBOV VLPs by both IM and ID injections. However, the levels of anti-GP antibodies were relatively low at about 3000 ng/ml for the IM group and 2500 ng/ml for the ID groups respectively. Further, analysis of sera neutralizing activity showed that these sera failed to reduce GP-pseudovirion infection by more than 50% at 1:100 dilution (data not shown).

**FIGURE 2:**
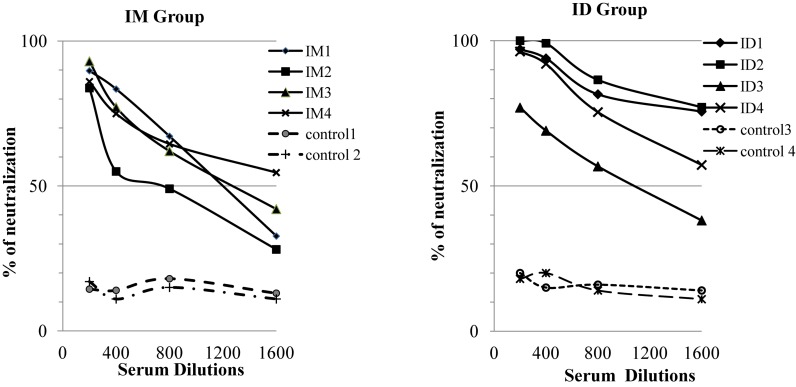
Neutralization of GP-mediated pseudovirion infection by sera from vaccinated guinea pigs. Neutralizing activity of immune sera collected after the third immunization was determined by a pseudovirion neutralization assay. Serial twofold dilutions of sera were inbubated with 500 pfu of GP-pseudotyped virus at 37°C for 1 h, the mixtures were then added to JC53 cells seeded in a 96-well plate and incubated at 37°C for 2 days. Neutralization was measured as decrease in luciferase expression compared to virus-only controls after 48 h as described in Materials and Methods. Results reported are the percentage of pseudovirion neutralization by samples from individual animals of each group at the indicated dilutions. Statistical comparison of serum neutralizing activities at each dilution was performed by a Student’s *t*-test, and was found to be not statistically significant (*p* > 0.05).

Based on these results, we decided to give one additional boosting immunization using 200 ug EBOV VLPs or SIVgag VLPs for the control group by IM or ID injection respectively as outlined in Figure 1A, and collected blood samples at 2 weeks after the third immunization to analyze the levels of antibodies against GP. As shown in Figure 1B (black columns), the levels of antibody responses against GP increased significantly to about 25000 ng/ml by more than eightfold for both IM and ID immunization groups after the additional boosting immunization. The neutralizing activity of sera from vaccinated guinea pigs was then determined by a pseudovirion neutralization assay. As shown in Figure 2, sera from guinea pigs vaccinated by EBOV VLPs via either IM or ID injection exhibited significant levels of neutralizing activity against EBOV GP mediated pseudovirion infection, with the average 50% neutralization titers reaching above 1:800 and 1:1600 dilutions for the IM and ID immunization groups respectively. However, analysis by a Student’s *t*-test showed that the differences in neutralizing activity between serum samples from guinea pigs in the IM and ID immunization groups at all four serum dilutions (1:200, 1:400, 1:800, and 1:1600) are not statistically significant (*p* > 0.05).

### Immunization Routes Affect the Profiles of Antibody Responses Against GP Induced by EBOV VLPs

**FIGURE 3:**
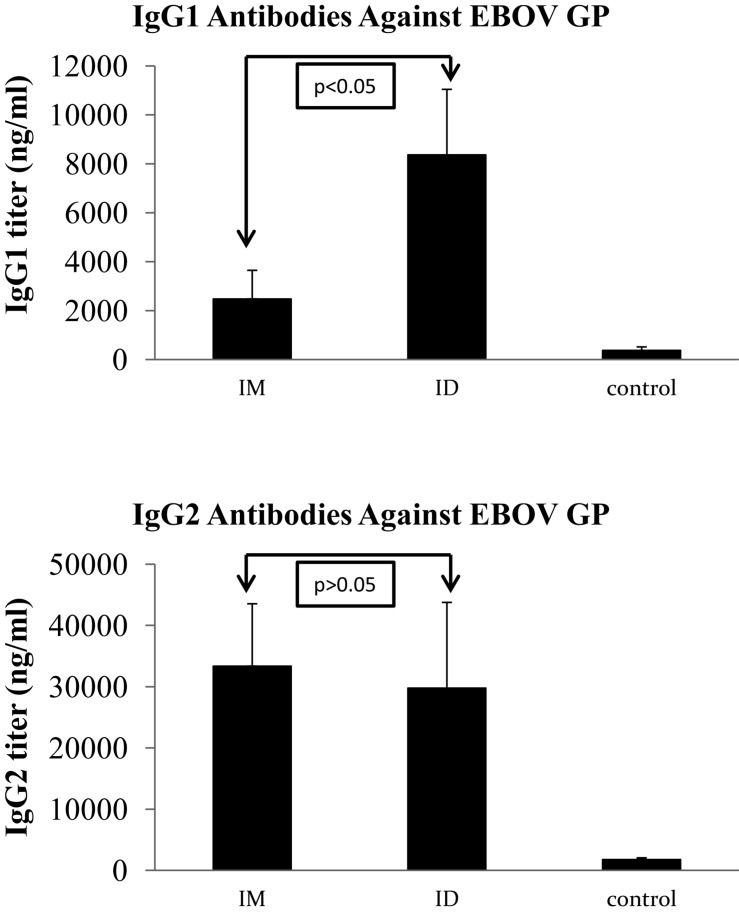
Analysis of IgG1 and IgG2 subclass antibody responses against GP induced by IM and ID injection of EBOV VLPs in guinea pigs. Guinea pig sera collected after the third immunization were analyzed for IgG1 and IgG2 subclass antibody responses against GP by ELISA using purified His-tagged GP as coating antigen. The concentrations of IgG1 and IgG2 antibodies against GP were determined from a standard curve and expressed as ng/ml of GP-specific antibodies in sera. Results reported are the means and standard deviations for samples from individual animals of each group. Statistical comparison of antibody levels against EBOV GP was performed by a Student’s *t*-test, and shown in each graph.

**FIGURE 4:**
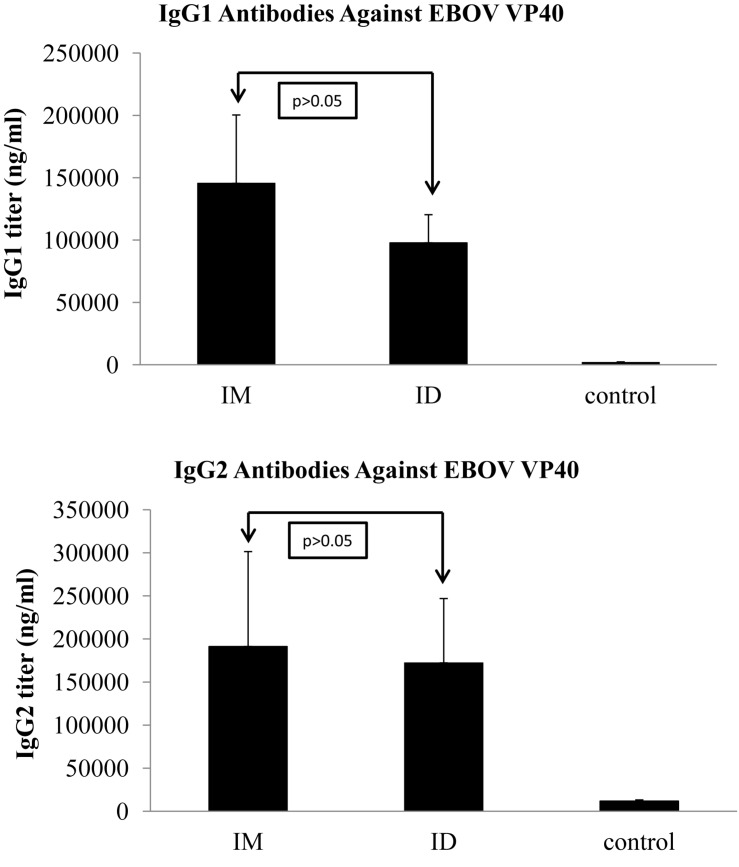
Induction of similar levels of IgG1 and IgG2 subclass antibody responses against VP40 induced by IM and ID injection of EBOV VLPs in guinea pigs. Guinea pig sera collected after the third immunization were analyzed for IgG1 and IgG2 subclass antibody responses against VP40 by ELISA using purified His-tagged VP40 as coating antigen. The concentrations of IgG1 and IgG2 antibodies against VP40 were determined from a standard curve and expressed as ng/ml of VP40-specific antibodies in sera. Results reported are the means and standard deviations for samples from individual animals of each group. Statistical comparison of antibody levels against EBOV GP was performed by a Student’s *t*-test, and shown in each graph.

We further characterized the levels of IgG1 and IgG2 subclass antibodies against GP induced by IM or ID immunization with EBOV VLPs for comparison. As shown in Figure 3, immunization by IM or ID injection of EBOV VLPs induced similar levels of IgG2 antibodies against GP in guinea pigs (*p* > 0.05). On the other hand, ID immunization by EBOV VLPs induced significantly higher levels of IgG1 antibodies against GP than IM immunization (*p* < 0.05). To investigate whether such differences might also be observed for other vaccine antigens in EBOV VLPs, we further determined the levels of IgG1 and IgG2 antibodies against EBOV matrix protein VP40 in EBOV VLPs for comparison. As shown in Figure 4, similar levels of both IgG1 and IgG2 antibodies against EBOV VP40 were induced by immunization through IM or ID injection of EBOV VLPs in guinea pigs (*p* > 0.05). Further, it is also notable that the levels of IgG1 antibodies against GP are only about one-third of the levels of IgG2 antibodies by ID immunization and about one-tenth of the levels of IgG2 antibodies by IM injection. In contrast, the levels of IgG1 and IgG2 antibodies against VP40 are similar in both IM and ID immunization groups. Taken together, these results show that the antibody profiles induced by IM and ID immunization of EBOV VLPs in guinea pigs are different only for the GP antigen but not for VP40.

**FIGURE 5:**
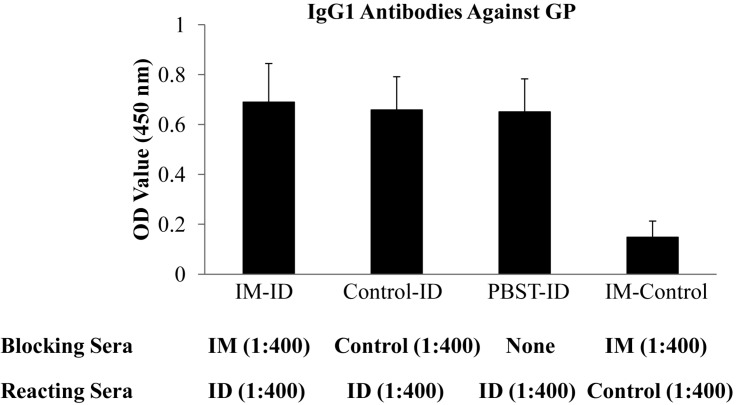
Binding of IgG1 from ID immunized guinea pigs to GP was not affected by pre-incubation with sera from IM immunized guinea pigs. Microtiter plates coated with His-tagged GP were first incubated with 1:400 dilutions of sera from guinea pigs vaccinated by IM injection of EBOV VLPs (IM-ID and IM-Control), IM or ID injection of SIVgag VLPs (Control-ID), or PBST with no sera (Control-ID) as indicated for 2 h at room temperature. The plates were then washed and incubated with 1:400 dilution of sera from guinea pigs vaccinated by ID injection of EBOV VLPs (IM-ID, Control-ID, and PBST-ID) or SIVgag VLPs (IM-Control) for 2 h at room temperature. The plates were washed again and the levels of IgG1 antibodies bound to each well were determined by incubating with horseradish peroxidase-labeled goat against guinea pig IgG1 antibodies and expressed as OD values for comparison. Results reported are the means and standard deviations for samples from paired animals of each group.

Based on the different levels of IgG1 antibodies against GP induced by ID and IM injection, we carried out blocking ELISA to determine whether the IgG1 and IgG2 antibodies against GP may target similar or different epitopes. Briefly, microtiter plates were coated with a limited amount of purified His-tagged GP protein at 0.05 ug per well. After blocking, the wells were treated with sera from guinea pigs vaccinated by IM injection of EBOV VLPs at 1:400 dilutions, a dilution that has been pre-determined to saturate binding of the amount of coated GP antigen. The controls were added with the same dilution of sera from control group guinea pigs or no sera as indicated. After incubating with the first antibody, the plate was washed and then added with sera from guinea pigs vaccinated by ID injection of EBOV VLPs or from control group guinea pigs at 1:400 dilutions as indicated, followed by addition of HRP-conjugated secondary antibody against guinea pig IgG1 to determine the levels of IgG1 antibodies bound to GP for comparison. The results in Figure 5 show that pre-incubation with sera from IM-vaccinated guinea pigs did not affect the binding of IgG1 antibodies in sera from ID-vaccinated guinea pigs to GP as compared to pre-incubation with sera from control guinea pigs or no sera. Further, without the addition of sera from ID-vaccinated guinea pigs, only low levels of IgG1 antibodies against GP in sera from IM-vaccinated guinea pigs were detected. These results suggest that the interaction of IgG1 antibodies to EBOV GP elicited by ID injection of EBOV VLPs is not affected by pre-blocking of the GP antigen by GP-specific antibodies from IM-vaccinated guinea pigs that are predominantly of the IgG2 subclass, suggesting that the IgG1 antibodies induced by ID immunization may target different epitopes in GP from those targeted by IgG2 antibodies induced by IM immunization with EBOV VLPs.

### Protection of Guinea Pigs Against Lethal EBOV Challenge

**FIGURE 6:**
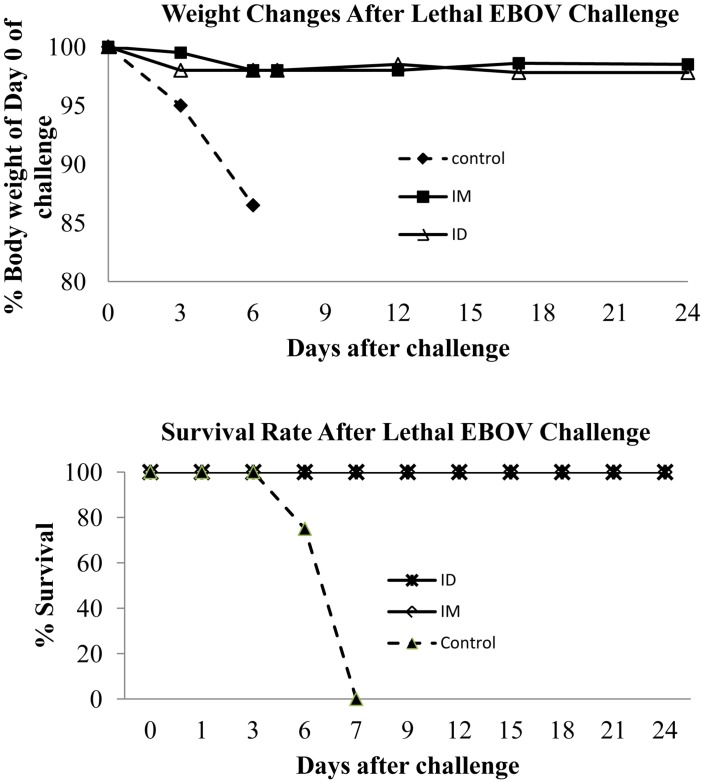
Guinea pigs immunized by EBOV VLPs are protected against a high dose lethal challenge. As outlined in Figure 1, guinea pigs were challenged by i.p. injection of 1000 pfu guinea pig-adapted EBOV at 22 weeks after the third immunization. Guinea pigs were monitored daily and weighed on indicated days post-challenge for recording of weight changes after challenge, and animals that exhibit substantial weight loss and signs of severe disease were sacrificed in accordance with IACUC guidelines.

After the third immunization, the guinea pigs were transferred to the Texas Biomedical Research Institute to evaluate the protective efficacy of EBOV VLP vaccination against lethal EBOV challenge in the ABSL-4 facility. As shown in Figure 1, vaccinated guinea pigs as well as the control group were infected by 1000 pfu of guinea pig-adapted EBOV at 22 weeks after the final immunization, and then observed daily for disease symptoms, weight changes, and body temperatures. As shown in Figure 6, all control group animals that received SIV Gag VLPs by either IM or ID injection succumbed to challenge with progressive weight losses and died by day 6 post-challenge. In contrast, all animals that had been vaccinated by EBOV VLPs by either IM or ID injection survived the challenge with no significant change in body weight over 24 days post-challenge. These results show that immunization by either IM or ID injection of EBOV VLPs effectively protected vaccinated animals against a high dose lethal challenge by guinea pig-adapted EBOV.

## Discussion

In the present study, we compared the effect of two different immunization routes, IM and ID injections respectively, on the immunogenicity and protective efficacy of insect cell-derived EBOV VLPs in the guinea pig model. Our results showed that EBOV VLPs administered via both IM and ID routes induced similar levels of antibody responses against EBOV GP and vaccinated were completely protected against a high dose lethal challenge by guinea pig-adapted EBOV. Of particular interest, we observed that while immunization with EBOV VLPs by IM or ID injection induced similar levels of IgG2 subclass antibodies against GP, the levels of IgG1 subclass antibodies against EBOV GP induced by ID immunization were significantly higher than the levels induced by IM immunizations. In contrast, immunization by both IM and ID routes induced similar levels of IgG1 and IgG2 antibody responses against VP40, the EBOV matrix protein in VLPs. Moreover, by further analyses, we observed that binding of IgG1 antibodies induced by ID injection of EBOV VLPs was not blocked by sera from IM-vaccinated animals, indicating that such IgG1 antibodies may react to different epitopes in GP from those recognized by antibodies induced by IM immunization, which are predominantly of the IgG2 subclass.

In addition to serving as a protective barrier, skin has also long been known to be an important part of the immune system (Bos and Kapsenberg, 1993). The first human vaccine, vaccinia, is administered by the ID route. Skin dermis is enriched with dermal DCs as well as Langerhans cells that are professional antigen presenting cells for immune surveillance (Flacher et al., 2006). In early studies, it has also been shown that influenza vaccines delivered by ID injection are safe and immunogenic in children (Rendtorff et al., 1959). However, due to the technical difficulty of ID injection by the conventional Mantoux method as well as the small volume of vaccines that can be delivered by this route, ID injection is not widely practiced for current vaccine delivery with the exception of the BCG vaccination against Tuberculosis. More recent studies showed that administration of influenza vaccines through ID immunization required reduced vaccine doses for eliciting similar levels of immune responses as IM delivery of influenza vaccines (Kenney et al., 2004; Chiu et al., 2007). In recent studies, new technologies are being development for vaccine delivery through the ID route. A new needle-syringe device that only exposed the tip of the needle has recently been designed for easier ID injection of influenza vaccines (Laurent et al., 2007; Holland et al., 2008), and this approach has now been approved for ID delivery of seasonal influenza vaccines. Moreover, microneedles are also under development for ID delivery of vaccines (Gill et al., 2014). Recent studies have shown that ID delivery of influenza vaccines using microneedles is superior to the conventional IM immunization approach for inducing antibody responses to confer long lasting protection against influenza virus infection in mice (Koutsonanos et al., 2012). We have in recent studies shown that ID delivery of adjuvanted EBOV GP or sGP protein subunit vaccines by microneedles was able to induce strong antibody responses against EBOV GP similarly to IM immunizations, and provided complete protection against lethal challenge in mice (Liu et al., 2018a, b). Our results from this study demonstrated that ID injection of EBOV VLPs is as effective as IM injection for eliciting immune responses in guinea pigs that are protective against lethal EBOV challenge. Further, by analyzing antibody responses after immunizations, we found that while similar levels of IgG2 antibodies against GP were induced by both IM and ID injections, ID immunization of EBOV VLPs induced significantly higher levels of IgG1 antibodies against GP than IM immunization. Moreover, our results from blocking ELISA studies show that the IgG1 antibodies induced by ID injection recognize different epitopes from the IgG2 antibodies induced by IM injection. These findings indicate that antigens delivered to dermal sites may be processed differently by local antigen presenting cells that leads to more comprehensive presentation of different epitopes for induction of immune responses. Future studies to determine the targets in GP recognized by IgG1 antibodies induced by ID immunization will reveal whether such antibodies may contribute to the control of virus infection either directly or in synergy with IgG2 antibodies and provide more effective protection against EBOV infection.

In contrast to antibody responses against GP, we found that the immunization by IM or ID injection of EBOV VLPs induced similar levels of both IgG1 and IgG2 antibodies against VP40, the EBOV matrix protein that directs the formation of VLPs. Further, the levels of IgG1 and IgG2 antibody subclasses against VP40 are also similar in animals vaccinated by EBOV VLPs through either IM or ID injection, whereas antibodies against GP are predominantly of the IgG2 subclass regardless of the immunization routes. These results indicate that induction of enhanced IgG1 antibody responses by ID immunization of EBOV VLPs is specific for the GP antigen but not the VP40 antigen, even when these antigens are delivered in the same VLP vaccine complex. The functional properties of guinea pig IgG1 and IgG2 antibodies are still poorly understood. However, in an early study, it was found that the IgG2 antibodies activate complement through the classical pathway whereas the IgG1 antibodies activate complement through the alternative pathway (Togashi and Tozawa, 1982). In this respect, it seems that guinea IgG1 and IgG2 antibodies resemble more closely to mouse IgG1 or IgG2a antibodies respectively (Lucisano Valim and Lachmann, 1991; Seino et al., 1993). As such, by comparing the IgG1/IgG2 antibody ratios induced by EBOV VLPs in guinea pigs in this study, it seems that the antibody response against GP is biased toward to the Th1 type whereas the antibody response against VP40 would suggest a balanced Th1/Th2 type. These results indicate that the Th1/Th2 paradigm as demonstrated by IgG1 and IgG2 antibody ratios after vaccination may be antigen specific, underscoring the need of more comprehensive analyses for understanding the mechanism as well as correlates of vaccine-induced immune response against virus infection.

## Conclusion

We show in this study that the EBOV VLPs produced by the recombinant baculoviruses from insect cells are able to provide effective protection against lethal challenge in the guinea pig model by both IM and ID immunizations. In comparison to IM immunization, ID immunization of EBOV VLPs was able to elicit enhanced IgG1 antibodies against epitopes in EBOV GP that were not recognized by the IgG2 antibodies induced by IM immunization. However, additional studies are needed to understand the underlying mechanism for this observation and to identify the targets recognized by the IgG1 antibodies induced by ID immunizations. Moreover, studies to determine the targets of the antibodies of different IgG subclasses induced by vaccination through different routes will further advance our understanding of the protection against EBOV infection by vaccine-induced immune responses. It will also be of interest to determine the effect of different adjuvant on the epitope breadth as well as subclass switching of antibody responses induced by ID immunizations in comparison with the conventional IM immunization approach. In particular, it will be of interest to determine if the use of an adjuvant will enhance or suppress induction of antibody responses to the subdominant epitopes in a vaccine antigen, and if subclass switching for dominant and subdominant epitopes will be similarly or differently affected by the use of an adjuvant. Moreover, optimization of EBOV VLPs or other subunit vaccines by ID immunization using novel microneedle vaccine delivery technologies will further reveal the potential of this vaccination approach for protection against infection by EBOV as well as other viral pathogens.

## Data Availability Statement

All datasets generated for this study are included in the article/supplementary material.

## Ethics Statement

The animal study was reviewed and approved by the Institutional Animal Care and Usage Committee (IACUC) of Emory University and the Institutional Animal Care and Usage Committee (IACUC) of TxBiomed Research Institute.

## Author Contributions

YL, LY, and ZW conducted production and characterization of EBOV VLP vaccines, immunization studies, and analyses of immune responses. RC, JN, HS, AT, and JP conducted EBOV challenge studies. LY, RWC, and CY contributed to experimental design and data analysis. YL, LY, and CY contributed to manuscript preparation.

## Conflict of Interest

The authors declare that the research was conducted in the absence of any commercial or financial relationships that could be construed as a potential conflict of interest.
